# Silver Nanoparticles Enhance Antimicrobial Efficacy of Antibiotics and Restore That Efficacy against the Melioidosis Pathogen

**DOI:** 10.3390/antibiotics10070839

**Published:** 2021-07-10

**Authors:** Sathit Malawong, Saengrawee Thammawithan, Pawinee Sirithongsuk, Sakda Daduang, Sompong Klaynongsruang, Pamela T. Wong, Rina Patramanon

**Affiliations:** 1 Department of Biochemistry, Faculty of Science, Khon Kaen University, Khon Kaen 40002, Thailand; sathitmalawong@hotmail.com (S.M.); sunshine.sc.bc19@gmail.com (S.T.); parbiochem@gmail.com (P.S.); somkly@kku.ac.th (S.K.); 2 Protein and Proteomics Research Center for Commercial and Industrial Purposes (ProCCI), Faculty of Science, Khon Kaen University, Khon Kaen 40002, Thailand; sakdad@kku.ac.th; 3 Division of Pharmacognosy and Toxicology, Faculty of Pharmaceutical Sciences, Khon Kaen University, Khon Kaen 40002, Thailand; 4 Department of Internal Medicine, University of Michigan Medical School, Ann Arbor, MI 48109, USA; ptw@umich.edu; 5 Michigan Nanotechnology Institute for Medicine and Biological Sciences, University of Michigan Medical School, Ann Arbor, MI 48109, USA

**Keywords:** melioidosis, *Burkholderia pseudomallei*, antimicrobial agent, silver nanoparticles, synergism, combination, multidrug resistance

## Abstract

Melioidosis is an infectious disease caused by Gram-negative bacillus bacteria *Burkholderia pseudomallei*. Due to the emerging resistance of *B. pseudomallei* to antibiotics including ceftazidime (CAZ), the development of novel antibiotics and alternative modes of treatment has become an urgent issue. Here, we demonstrated an ability to synergistically increase the efficiency of antibiotics through their combination with silver nanoparticles (AgNPs). Combinations of four conventional antibiotics including CAZ, imipenem (IMI), meropenem (MER), and gentamicin sulfate (GENT) with starch-stabilized AgNPs were tested for their antibacterial effects against three isolates of *B. pseudomallei*. The combination of each antibiotic with AgNPs featured fractional inhibitory concentration (FIC) index values and fractional bactericidal concentration (FBC) index values ranging from 0.312 to 0.75 µg/mL and 0.252 to 0.625 µg/mL, respectively, against the three isolates of *B. pseudomallei*. The study clearly showed that most of the combinatorial treatments exhibited synergistic antimicrobial effects against all three isolates of *B. pseudomallei*. The highest enhancing effect was observed for GENT with AgNPs. These results confirmed the combination of each antibiotic with AgNPs restored their bactericidal potency in the bacterial strains that had previously been shown to be resistant to the antibiotics. In addition, morphological changes examined by SEM confirmed that the bacterial cells were severely damaged by combinations at the FBC level. Although bacteria produce fibers to protect themselves, ultimately the bacteria were killed by the antibiotic–AgNPs combinations. Overall, these results suggest the study of antibiotic–AgNPs combinations as an alternative design strategy for potential therapeutics to more effectively combat the melioidosis pathogen.

## 1. Introduction

Melioidosis is an infectious disease caused by Gram-negative bacillus bacteria *Burkholderia pseudomallei*; this organism is an important causative agent of septicemia and is community-acquired. It is believed to be vastly underreported, with 165,000 cases worldwide and the fatality rate of approximately 89,000 deaths per year [1]. The incidence of melioidosis is the highest in Southeast Asia and northern Australia, with the fatality rate of 40% in northeast Thailand and 19% in Australia [2,3]. Currently, no licensed vaccine against melioidosis has been established for clinical use. Melioidosis has been dubbed “the great imitator” as it presents with great clinical diversity, and several of its symptoms are often confounded with those of other diseases, such as tuberculosis [4]. Furthermore, melioidosis can also sometimes be asymptomatic. Altogether, these features make the disease difficult to diagnose.

The selection of antibiotics for the treatment of melioidosis is limited due to the bacteria’s resistance to several commonly prescribed antibiotics, including aminoglycosides, fluoroquinolone compounds, and many β-lactam antibiotics [5,6]. Ceftazidime (CAZ), a third-generation antibiotic of the cephalosporin family, is recommended as the first-line therapy for the treatment of severe melioidosis [7,8,9]. CAZ works by interfering with bacterial cell wall synthesis. Carbapenem antibiotics, such as imipenem and meropenem, have also shown potent activity against *B. pseudomallei* [10,11]. Although the resistance of *B. pseudomallei* to CAZ is rare, it has been demonstrated to occur both in vitro and in vivo [6,12,13]. Moreover, as with any antibiotic, repeated exposure to the drug through increased use elevates the risk of developing bacterial resistance over time. The increasing prevalence of antibiotic resistance has become a serious public health problem worldwide, and alternative therapies that can overcome resistance and prevent future resistance are urgently needed. One approach to controlling bacterial resistance is through using a combination of antibiotics with other agents that increase the efficacy of the antibiotic. These agents include other antibiotics [14,15,16], antimicrobial peptides [17,18], plant extracts [19,20], and nanoparticles [21,22].

Nanoparticles are of great interest for researchers as they have unique physical, chemical, and electrical properties that differ from bulk materials. Such properties are the result of the shape and size of the nanoparticles which have a high surface area-to-volume ratio due to their small size. Among the nanoparticles, silver nanoparticles (AgNPs) have attracted the most attention because they have broad-spectrum efficacy against several microorganisms, including bacteria, fungi, and viruses, with low cytotoxicity to mammalian cells [23,24]. AgNPs have multiple modes of action that lead to bacterial cell killing, including the rupture of the bacterial cell membrane through AgNP adherence and the penetration of AgNPs into the cell and the nucleus, resulting in binding interactions with proteins and DNA and leading to ROS production and subsequently to cell death [25,26]. Due to the nonspecific nature of these mechanisms, AgNPs do not place selective pressure on bacteria and have a much lower risk for the development of resistance compared to conventional antibiotics.

Combinations of AgNPs with antibiotics (e.g., with ampicillin, gentamicin, and vancomycin) have been reported to have synergistic antibacterial effects toward both nonresistant and resistant strains [27,28]. In addition, several studies examining the synergistic activity of AgNPs in combination with other antibiotics have been reported: combinations of AgNPs and cefotaxime or CAZ, MER, ciprofloxacin, or GENT strongly enhanced antibacterial activity against multidrug-resistant β-lactamase- and carbapenemase-producing *Enterobacteriaceae* [29]. AgNPs with enoxacin, kanamycin, neomycin, or tetracycline showed greater bactericidal efficiency toward the drug-resistant bacteria *Salmonella typhimurium* [30]. Combinations of AgNPs with chloramphenicol or kanamycin resulted in synergistic bactericidal activity toward *Pseudomonas aeruginosa*, a virulent species sharing a common ancestry with *B. pseudomallei* [31,32]. In this study, we investigated the synergistic antimicrobial effects of antibiotics and AgNPs against *B. pseudomallei* which had not been reported before. AgNPs were combined with four types of antibiotics against *B. pseudomallei* from three clinical isolates. The synergistic antibacterial effects were evaluated by measuring the FIC indices and the FBC indices which were obtained by plate counts using the microdilution checkerboard method. The growth inhibition curve and the kinetics of killing were assessed by UV–Vis spectroscopy and counting colony-forming units. Moreover, we compared the changes in bacterial cell morphology between the treatment with individual and combination therapies using scanning electron microscopy (SEM).

## 2. Results

### 2.1. Characterization of Silver Nanoparticles

**Figure 1:**
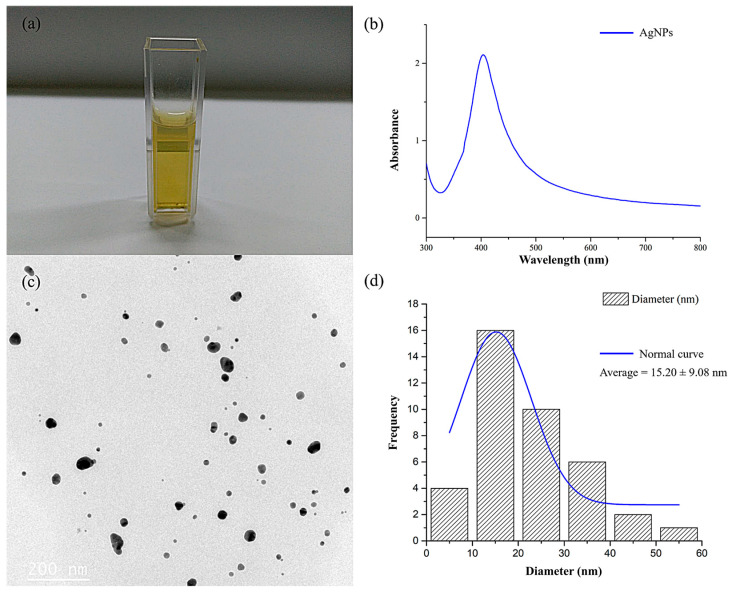
Physicochemical characteristics of the AgNPs: (**a**) AgNP solution; (**b**) UV–Vis spectrum absorption of the AgNPs; (**c**) TEM image of the AgNPs; and (**d**) size distribution of the AgNPs based on a TEM image.

The colloidal starch-stabilized AgNPs (at a concentration of 1 mg/mL) used in this study were purchased from a commercial source. The solution was diluted to 100 µg/mL in deionized water, resulting in a yellowish solution (Figure 1a). The AgNPs were characterized by UV–Vis spectroscopy and TEM to observe their size, shape, and homogeneity. The absorbance spectra showed a single strong peak at 404 nm, which indicated the presence of spherical AgNPs (Figure 1b) [33]. A peak near 400 nm corresponds to smaller nanospheres, while larger spheres exhibit increased scattering and have broadened peaks shifted towards longer wavelengths as shown in previous reports [34,35]. A TEM micrograph confirmed that the particles had a spherical shape and demonstrated monodispersity (Figure 1c). The AgNPs had an average size of 15.20 ± 9.08 nm in diameter, as calculated using the Image J software (Figure 1d).

### 2.2. Antimicrobial Susceptibility

Individual antimicrobial activities of CAZ, IMI, MER, GENT, and AgNPs against *B. pseudomallei* were determined. The first three are conventional antibiotics used in melioidosis treatment, while *B. pseudomallei* is normally resistant to the GENT antibiotic. As shown in Table 1, the MICs of CAZ, IMI, MER, and GENT were in the ranges of 4–128 µg/mL, 0.5–1 µg/mL, 2 µg/mL, and 16–64 µg/mL, respectively. As expected, *B. pseudomallei* 1026b and H777, but not *B. pseudomallei* 316c, were susceptible to CAZ. All three isolates were susceptible to IMI and MER and completely resistant to GENT. According to a previous report, the *B. pseudomallei* antibiotic breakpoint used for in vitro susceptibility testing of CAZ was 32 µg/mL, and that of IMI, MER, and GENT was 8 µg/mL [6]. Of these, IMI and MER were the most effective for the treatment of the three isolates of *B. pseudomallei*, while GENT had the lowest effectiveness.

CAZ, IMI, MER, and GENT had MBCs in the ranges of 64–512 µg/mL, 1–2 µg/mL, 4 µg/mL, and 256–512 µg/mL, respectively (Table 1). The MBCs of CAZ and GENT were much higher than the antibiotic breakpoints used for the *B. pseudomallei* susceptibility testing. This indicates that all three isolates of *B. pseudomallei* are difficult to kill by CAZ and GENT, but not by IMI and MER.

The MICs and MBCs of AgNPs against the three isolates of *B. pseudomallei* tested were in the ranges of 8–16 µg/mL and 16–32 µg/mL, respectively. The MBC values of AgNPs are lower than those of CAZ and GENT but higher than those of IMI and MER. In addition, AgNPs had a higher antimicrobial activity against the CAZ-nonresistant isolate than against the CAZ-resistant isolates.

### 2.3. Synergistic Antibacterial Effects

**Figure 2:**
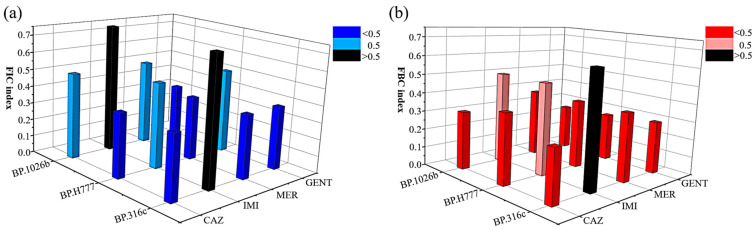
Three-dimensional graphs showing the synergistic antibacterial activity of antibiotics with AgNPs. Comparison of the FIC (**a**) and FBC (**b**) indexes for each of the antibiotics when combined with AgNPs against the three isolates of *B. pseudomallei*. Synergy was defined as the FIC or FBC index values ≤ 0.5; antagonism was defined as the FIC or FBC index values >4; and indifference was defined as the FIC or FBC index values > 0.5 and ≤ 4. The data represent three independent experiments that all gave identical values, and no standard deviation was observed.

FIC and FBC indices (FICI and FBCI) are commonly used to define the synergistic effects of agents for the inhibition and killing of bacteria [36]. Synergism is defined as the FIC or FBC index values ≤ 0.5, and indifference is defined as the FIC or FBC index values > 0.5 and ≤ 4. The FIC indices of CAZ, IMI, MER, and GENT in combination with AgNPs against the three isolates of *B. pseudomallei* are shown in Figure 2a. The blue and light blue bars in the graph represent combinations with an FIC index ≤ 0.5, while the black bars in the graph represent combinations with an FIC index > 0.5. The FIC indices of CAZ, MER, and GENT in combination with AgNPs indicate that these antibiotics have a synergistic inhibitory effect (FIC index ≤ 0.5) with AgNPs against *B. pseudomallei*. The combination of IMI with AgNPs, however, showed no synergism against *B. pseudomallei* 1026b and 316c (black bar graph, FIC index > 0.5). This indicates that cotreatment with IMI and AgNPs does not improve the antibacterial efficacy against *B. pseudomallei* 1026b and 316c.

Figure 2b shows the FBC indices of CAZ, IMI, MER, and GENT in combination with AgNPs. The red and pink bars in the graph represent FBC indices ≤ 0.5, while the black bars represent FBC indices > 0.5. The FBC indices of CAZ, IMI, MER, and GENT in combination with AgNPs are ≤ 0.5, which reveals that these agents have synergistic bactericidal effects against *B. pseudomallei* in the three isolates, with the exception of IMI–AgNPs against *B. pseudomallei* 316c, which showed indifference.

Table 2 and Table 3 show the lowest antibiotic concentrations that have a synergistic effect with AgNPs. The MICs of the antibiotics alone, AgNPs alone, or of the combinations of antibiotics with AgNPs are presented in Table 2. The concentrations of CAZ, IMI, MER, and GENT in combination with AgNPs that inhibited bacterial growth were in the ranges of 1–16 µg/mL, 0.25–0.5 µg/mL, 0.5 µg/mL, and 2–16 µg/mL, respectively. The concentrations of AgNPs in combination with each antibiotic were in the range of 2–4 µg/mL. We demonstrated that the combination of antibiotics and AgNPs allows the use of lower antibiotic concentrations to achieve equivalent antimicrobial efficiency. The MIC concentrations of combinations with CAZ decreased up to 4–8-fold, with IMI—up to 2–4-fold, with MER—up to fourfold, and with GENT—up to 4–16-fold when compared with the antibiotics alone. Furthermore, the MIC concentrations of combinations with AgNPs decreased up to 2–4-fold compared with AgNPs alone.

Likewise, the MBCs of the antibiotics alone, AgNPs alone, or the antibiotics with AgNPs are presented in Table 3. The bactericidal concentrations of CAZ, IMI, MER, and GENT in combination with AgNPs were in the ranges of 4–16 µg/mL, 0.25–0.5 µg/mL, 1 µg/mL, and 1–16 µg/mL, respectively. The concentration of CAZ in combination decreased up to 4–32-fold when compared with CAZ alone, of IMI, MER, and GENT—up to 2–4-fold, fourfold, and 32–512-fold, respectively. Furthermore, the MBC concentrations of AgNPs in combination were in the range of 2–8 µg/mL, i.e., decreased up to 4–16-fold compared with AgNPs alone. As a result, the decrease in the concentration of the antibiotic needed to achieve the same inhibition activity in combination with AgNPs was in the following order: GENT > CAZ > MEM > IMI. The greatly decreased MBCs for the antibiotic–AgNPs combinations imply that the combination increased the susceptibility of *B. pseudomallei* to antibiotics.

### 2.4. Growth Curve of B. pseudomallei in the Presence of a Combination

**Figure 3:**
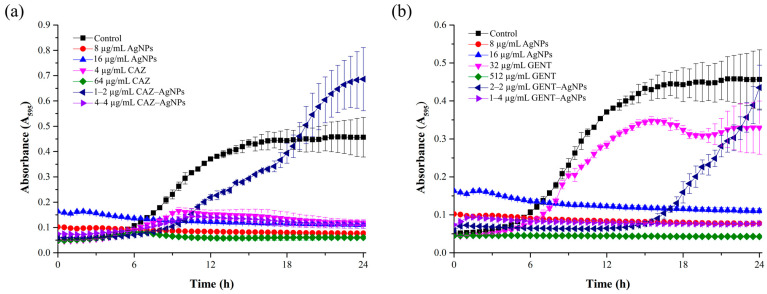
Bacterial growth curves of *B. pseudomallei* in the presence of different concentrations of AgNPs alone, the antibiotics alone, an antibiotic–AgNPs combination ((**a**) CAZ; (**b**) GENT). The incubation of agents and bacteria was measured at 630 nm every 30 min for 24 h. The data represent the mean values ± SD (error bars) from two independent experiments carried out in triplicate.

The growth curve of the bacteria treated with the antibiotics alone and in combination is shown in Figure 3. As shown in Figure 3a, the growth curve of *B. pseudomallei* in the presence of CAZ alone, AgNPs alone, and CAZ–AgNPs in combination exhibited inhibited bacterial growth throughout 24 h. Only at 1–2 µg/mL of the CAZ–AgNPs combination (FIC) the growth of the bacteria was observed after 9 h. As a result, the data indicated that AgNPs alone, CAZ alone, and 4–4 µg/mL of CAZ–AgNPs (FBC) could reduce the bacterial intensity compared with the untreated control at 24 h (*p* < 0.001). In the same way, in Figure 3b, the bacteria treated with GENT alone and with AgNPs alone and in combination exhibited inhibited bacterial growth throughout 24 h. However, at 32 µg/mL of GENT alone (MIC) and 2–2 µg/mL of GENT–AgNPs (FIC), an increasing number of the bacterial cells was observed after 6 h and 15 h, respectively. These results indicated that AgNPs alone, 512 µg/mL of GENT (MBC), and 1–4 µg/mL of GENT–AgNPs (FBC) were able to reduce the bacterial intensity compared with the untreated control at 24 h (*p* < 0.001).

### 2.5. Time-Dependent Killing Efficiency of Combinations

**Figure 4:**
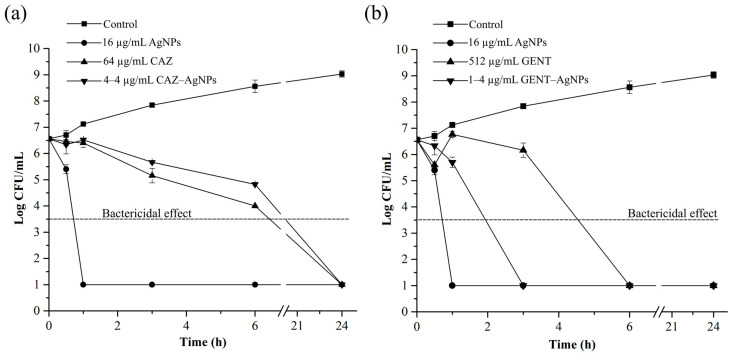
Changes in time-dependent killing efficiency of each antibiotic after addition of AgNPs. *B. pseudomallei* were treated with CAZ alone, AgNPs alone, and the CAZ–AgNPs combination (**a**) and with GENT alone, AgNPs alone, and the GENT–AgNPs combination (**b**). Bacteria were incubated at the MBC or FBC of each agent at 37 °C. The solutions were sampled at 0, 0.5, 1, 3, 6, and 24 h for colony count. A decrease in the bacterial colony ≥ 3 log_10_ was defined as the bactericidal effect. The data represent the mean values ± SD (error bar) from two independent experiments each carried out in triplicate.

The kinetics of bactericidal activity of the antibiotics alone, AgNPs alone, and antibiotic–AgNPs combinations against *B. pseudomallei* are presented in Figure 4. As shown in Figure 4a,b, AgNPs alone, CAZ alone, and GENT alone were able to reduce the bacterial colonies by more than 3 log_10_ when compared to the initial inoculum (*p* < 0.001). These results of AgNPs alone, CAZ alone, and GENT alone indicated the bactericidal effect within 1 h, after 6 h and within 6 h, respectively. The combinations of CAZ with AgNPs and GENT with AgNPs reduced the number of the bacterial cells by more than 3 log_10_ when compared to the initial inoculum (*p* < 0.001), indicating the bactericidal effect after 6 h and within 3 h, respectively. AgNPs alone exhibited the fastest bactericidal effect, followed by the GENT–AgNPs combination and the CAZ–AgNPs combination. Moreover, the combination of GENT with AgNPs exhibited a faster bactericidal activity than GENT alone, whereas CAZ with AgNPs exhibited bactericidal activity at the same time as CAZ alone.

### 2.6. Cell Morphological Change

**Figure 5:**
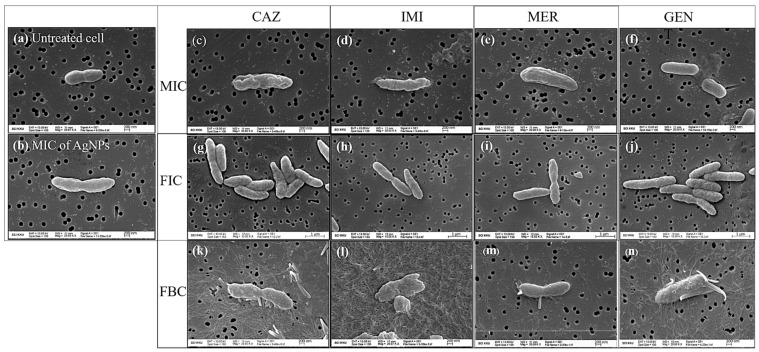
Scanning electron micrographs of *B. pseudomallei* 1026b: untreated (**a**) or treated with the MIC of AgNPs (**b**), CAZ (**c**), IMI (**d**), MER (**e**), or GENT (**f**). SEM images of 1026b treated with the FIC of AgNPs with CAZ (**g**), IMI (**h**), MER (**i**), or GENT (**j**) for 5 h. SEM images of 1026b treated with the FBC of AgNPs with CAZ (**k**), IMI (**l**), MER (**m**);,or GENT (**n**) for 1 h.

To evaluate the morphology of the bacterial cells under different treatment conditions, we observed the morphological changes of *B. pseudomallei* 1026b treated with CAZ, IMI, MER, or GENT alone or in combination with AgNPs using SEM (Figure 5). The SEM images showed that the untreated control cells appeared intact, plump, and typically rod-shaped, with a smooth exterior (Figure 5a). In contrast, the bacterial cells exposed to antibiotics alone or in combination with AgNPs at the MIC and FIC levels exhibited loss of membrane integrity, especially in the case of treatment with beta-lactam antibiotics (CAZ, IMI, MER) at the MIC level. The cell walls became loose and porous, distorted from their normal shape, or even ruptured (Figure 5b–j). Furthermore, we noticed that the treatment of the bacteria with the antibiotics alone and in combination with AgNPs resulted in more elongated cells compared to the control cells.

We further observed the morphology of the bacteria treated with combinations of the antibiotics with AgNPs at the FBC (Figure 5k–n). The results clearly show that the bacterial cells were more severely damaged with this combination than with those at the MIC and FIC. At the FBC, microscopic analysis of the bacterial cells revealed gross leakage and holes on the outer surface, with a bulgy, disfigured, and fragmented shape. Surprisingly, under these conditions, the bacterial cells produced a substantial amount of fiber which appeared within 1 h.

## 3. Discussion

In this study, we demonstrated the ability to synergistically increase the efficiency of antibiotics through their combination with AgNPs to overcome antibiotic-resistant bacteria. The starch-stabilized AgNPs solution was obtained from the manufacturer (Prime Nanotechnology Co., Ltd., Bangkok, Thailand) with the average size of 15.20 ± 9.08 nm in diameter; previous studies have indicated that AgNPs of 10–20 nm in diameter have antimicrobial efficiency against *B. pseudomallei* [37]. The antibiotics and AgNPs alone were then evaluated for their antimicrobial susceptibility against the three isolates of *B. pseudomallei*. It can be observed that the antibiotics and AgNPs have different antimicrobial efficiency against *B. pseudomallei* (Table 1). Antimicrobial susceptibility of the antibiotics and AgNPs alone showed that *B. pseudomallei* resisted CAZ and GENT, but it was still susceptible to IMI and MER. These results are the same as in a previous study that reported that *B. pseudomallei* was susceptible to IMI and MER but extremely resistant to GENT [38]. We found that AgNPs have a strong antimicrobial effect against these bacteria. The MICs and MBCs of AgNPs were lower than those observed for *B. pseudomallei* in a previous study, which reported the MICs and MBCs of AgNPs against *B. pseudomallei* in these three isolates in the ranges of 32–48 µg/mL and 96–128 µg/mL, respectively [37]. This was because some properties of the AgNPs, such as size and shape, were slightly different. The smaller the size, the higher the antimicrobial activity. These differences can cause uncertainty in biological activity [39].

After an antimicrobial susceptibility test, a synergism assay was performed. Combinations of each antibiotic with AgNPs revealed that all the antibiotics, when in combination with AgNPs, have strong synergistic effects on inhibiting the growth and killing (FICI and FBCI ≤ 0.5) of *B. pseudomallei* in the three isolates. AgNPs enhanced antibacterial activity of all the antibiotics at the concentrations far below the MIC and MBC of the antibiotics alone (Table 2 and Table 3). In particular, GENT with AgNPs clearly showed a large decrease in the dose of GENT needed to kill the bacteria (decrease in the MBC concentration up to 512-fold). AgNPs restored the bactericidal activity of GENT, an inactive antibiotic for *B. pseudomallei*. Our results are similar to many reports that have shown combinations of GENT with AgNPs to have bactericidal activity against *S. epidermidis*, *P. aeruginosa*, and *A. baumannii* [40,41]. Since no previous reports have been made on the use of combinations of CAZ, IMI, MER, or GENT with AgNPs against *B. pseudomallei*, we could only compare the combinations with the results reported for other Gram-negative bacteria. Our results are similar to those of Panácek et al. AgNPs showed strongly enhanced bactericidal activity and restored bactericidal activity of inactive antibiotics (CAZ, MER, and GENT) against both antibiotic-susceptible and multidrug-resistant *Enterobacteriaceae* [42]. Another study showed a strong synergistic antibacterial effect of the IMI–AgNPs combination against IMI-resistant *K. pneumonia* [29].

These increases in antimicrobial activity could be due to several reasons for the possible mechanism between AgNPs and the beta-lactam antibiotics (CAZ, IMI, and MER); a previous study of a beta-lactam antibiotic (amoxicillin) indicated that the synergistic effect may be caused by a bonding reaction between the antibiotic and AgNPs. This suggests that the concentration of antimicrobial groups at particular points on the cell surface may increase the severity of damage to the bacterial cell [43]. Another study demonstrated that the synergistic effect may be the action of the “AgNPs’ drug carrier.” Moreover, membrane phospholipids and glycoproteins have been targeted by hydrophobic AgNPs, with amoxicillin being transported to the cell surface to damage the cell [44,45].

For GENT with AgNPs, there was a previous report that suggested that *Staphylococcus aureus* can be killed through the interaction of GENT and AgNPs. Hydroxyl and amide groups of GENT easily react with AgNPs, and they can then deliver the drug to the cell [46]. Another previous report studied the effects of combining GENT with AgNPs against *S. aureus*, *E. coli*, and GENT-resistant *E. coli*; a dual role for GENT was found in which it increased the dissolution of AgNPs and facilitated the attachment of AgNPs onto the surface of the bacteria, thereby enhancing the antibacterial activity of AgNPs [27].

However, among all the conditions tested, only one combination showed no synergistic effect: IMI in combination with AgNPs against *B. pseudomallei* 1026b and 316c. This result could imply that the action of antibiotics with AgNPs depends on the bacterial isolates because of the cell membrane component of each *B. pseudomallei* isolate [47,48]. In a similar study, a combination of antibiotics including ampicillin, chloramphenicol, or kanamycin with AgNPs showed differences in activity between the two isolates of *Escherichia coli* tested. The combinations showed synergism and partial synergism in inhibiting and killing *E. coli* ATCC 43895 and *E. coli* ATCC 25922, respectively [31].

As mentioned above, based on these results, a combination of each antibiotic with AgNPs increased the susceptibility of *B. pseudomallei*, confirming that combinations of antibiotics with AgNPs can lead to an increase in their antimicrobial activity. Next, we observed the bacterial growth curve and kinetic killing of beta-lactam (CAZ) or aminoglycoside (GENT) combinations with AgNPs to evaluate bacterial growth inhibition and the rate at which bacteria are killed by the combination. The results of the bacterial growth curve imply that the combination of CAZ or GENT with AgNPs can inhibit and slow down the bacterial growth in a dose-dependent manner. The combination at the FIC level of CAZ or GENT with AgNPs showed bacterial regrowth because these concentrations could not eradicate all the bacterial cells unlike the FBC.

The time-dependent killing efficiency of the combinations displayed improving kinetic killing of GENT but not of CAZ. AgNPs improved the bactericidal activity of GENT, which caused the complete killing of bacterial cells within 3 h, in a shorter time than with GENT alone. On the other hand, AgNPs did not improve the time-dependent killing efficiency of CAZ in the combination, with the complete killing after 6 h, the same as with CAZ alone. These results demonstrate the different mechanisms of action of the beta-lactam and aminoglycoside combinations with AgNPs. The synergism of GENT and AgNPs might occur through the interaction of GENT and AgNPs as mentioned earlier [46], which can then deliver the drug to the cells, resulting in the GENT–AgNPs combinations presenting a shorter time than GENT alone but a longer time than AgNPs alone. For the CAZ–AgNPs combination, a previous study showed that the synergism of beta-lactam antibiotics with AgNPs could be through an inhibition of beta-lactamase by AgNPs at sub-MIC; hydrolysis of the antibiotics does not occur, therefore the antibiotics remain effective against bacteria [29]. This corresponds to the time-dependent killing efficiency of CAZ–AgNPs that showed complete killing after 6 h, the same as for CAZ alone.

The morphology of the bacterial cells under different treatment conditions was analyzed to confirm death and morphological changes of *B. pseudomallei*. The results showed distinct features for the MIC. Beta-lactams have a mechanism of action on bacterial cell wall inhibition, resulting in the cells clearly shrinking due to loss of membrane integrity. At the FIC, all the conditions were similar due to the AgNPs added. The bacterial cells were more severely damaged at the FBC than at the MIC and FIC because at the MIC and FIC, the agents could only inhibit bacterial growth, whereas at the FBC, the bacteria were killed. Moreover, at the FBC, the bacterial cells exhibited massive damage, especially in the case of the GENT–AgNPs combination which produced holes in the bacterial cells. These might be caused by different mechanisms for each antibiotic because beta-lactam inhibits cell wall synthesis while GENT inhibits bacterial protein synthesis. Moreover, observing the fibers produced under such harsh conditions at the FBC could be exopolysaccharides, or EPS (also known as extracellular polysaccharides), which are part of the biofilm found in the extracellular medium surrounding the bacteria [49]. Normally, *B. pseudomallei* can produce a biofilm to protect them from proximal unsuitable environments, but it can also produce large biofilms in severe conditions [50,51]. The EPS in biofilms are a variety of macromolecules, including proteins, DNA, lipids, and polysaccharides (the main structural component). Polysaccharides are produced first during biofilm production to allow the bacterial cell to adhere to the surface. Subsequently, the bacteria proceed to create suitable conditions for survival that protect them against the dangerous environment [52,53].

We demonstrated that the FBC is a severely stressful level that causes *B. pseudomallei* to produce large amounts of fibers within 1 h to protect the cells. Several studies have shown that at subminimal inhibitory concentrations (sub-MIC; 1/2 MIC), an antibiotic can establish stress conditions to bacteria that stimulate bacterial biofilm formation in vitro [54,55,56]. As shown in Figure 5l for an IMI–AgNPs combination, this condition evidently exhibited fiber production. IMI concentration for bactericidal synergism (FBC) is 0.5 μg/mL which is sub-MIC of IMI alone (1 μg/mL) against *B. pseudomallei* 1026b, the same as for MER in Figure 5m; bactericidal synergism occurs at 1 μg/mL that is sub-MIC of MER alone (2 μg/mL). These results are also similar to the report of Pier Carlo Braga et al. that CAZ induces widespread production of filamentous forms at levels ranging from 1/2 to 1/8× MIC [57]. Numerous studies have reported that antibiotics such as beta-lactams that induce cell lysis can stimulate biofilm formation [58,59]. In the same way, GENT and other aminoglycosides have significant biofilm formation at the sub-MIC as well [60,61]. Meanwhile, much research has proven that the sub-MIC of AgNPs can stimulate biofilm development [62,63]. These phenomena can be explained by one of the mechanisms of action of AgNPs, formation of reactive oxygen species (ROS), which cause oxidative stress. As shown in Figure 5k,l,n, a combination of AgNPs at the sub-MIC (4 μg/mL) with each antibiotic showed fiber production. Therefore, the production of bacterial fiber at the FBC could be an effect of the antibiotic or AgNPs at the sub-MIC.

Previous studies have indicated that the biofilm of *B. pseudomallei* is not a virulence factor, but is associated with melioidosis relapse because of its facilitation of antibiotic resistance development [64,65]. Importantly, high levels of EPS in biofilms have been demonstrated to inversely correlate with the ability of antibiotics and nanoparticles to penetrate *B. pseudomallei* [66]. It is possible that the fiber production observed at the FBC may be a relapsing factor that causes bacterial resistance to a combination of antibiotics with AgNPs in the future. However, although the bacteria tried to produce fiber to protect the cells, in the end, they were killed by the antibiotic–AgNPs combination. This might be because AgNPs can penetrate the biofilm and because of the synergism effect to enhance anti-biofilm activities of AgNPs with antibiotics, as in the report of Gurunathan et al. [67]. However, to extend understanding of this phenomenon, more elaborate experimental evidence could be required in future works.

Overall, we showed that most of the examined antibiotics have a synergistic effect with AgNPs. The advantage of combinations improves both concentration and time to overcome bacteria that could reduce side effects by enabling the use of a lower dose of antibiotics and AgNPs. The reduction of time to kill the bacteria might be useful to reduce the risk of bacterial resistance induction. Moreover, we noticed the combinations of antibiotics with AgNPs at the sub-MIC that might trigger EPS production by bacteria. However, the aforementioned sub-MIC of the antibiotics and AgNPs in combination could be optionally selected to avoid an inapplicable condition.

## 4. Materials and Methods

### 4.1. Materials and Cell Culture

The antibiotics were purchased from their respective manufacturers and dissolved according to the recommendations. The antibiotics tested were CAZ (Reyoung Pharmaceutical Co., Ltd., Shandong, China), IMI (JW Pharmaceutical Corporation, Seoul, Korea), MER (Siam Bheasach Co., Ltd., Bangkok, Thailand), and GENT (Sigma-Aldrich, St. Louis, MO, USA). Starch-stabilized AgNPs were obtained by our collaborative company Prime Nanotechnology Co., Ltd. (Bangkok, Thailand) with a stock concentration of 1000 mg/L. For AgNP solution preparation in our experiments, 1 mg/mL as a stock solution was prepared in sterile deionized water. AgNPs were then serially diluted by twofold dilution in the range of the final concentration of 1–512 µg/mL, then kept at room temperature until use. The following isolates were used in this study: *B. pseudomallei* 1026b (CAZ-nonresistant isolate), *B. pseudomallei* H777 (moderately CAZ-resistant isolate), and *B. pseudomallei* 316c (highly CAZ-resistant isolate). They were provided by the Melioidosis Research Center, Khon Kaen University. All the strains were isolated from the blood of patients [68,69,70]. These bacteria were stored at −70 °C in 20% glycerol in microcentrifuge tubes until use. The bacteria were streaked on Ashdown’s medium (a selective culture medium for the isolation and characterization of *B. pseudomallei*) and then cultured at 37 °C for 48–72 h. The colonies were picked and inoculated in 5 mL Mueller Hinton broth (MHB) at 37 °C overnight and then subcultured in 5 mL of the same medium at 37 °C in a 180 rpm shaker/incubator for 3 h to yield a mid-logarithmic growth phase culture [71].

### 4.2. Preparation and Characterization of Silver Nanoparticles

Starch-stabilized AgNPs with diameters of 10–20 nm were obtained from Prime Nanotechnology, Bangkok, Thailand. The samples were resuspended in deionized water at the concentration of 1 mg/mL. The UV–Vis spectra of the AgNPs were recorded using a SpectraMax M5 fluorescence microplate reader (Molecular devices, San Jose, CA, USA). The dimensions of the AgNPs were confirmed using a transmission electron microscope (FEI/TECNAI G2 20, FEI Company, Hillsboro, OR, USA) operating at 200 kV. The sizes of the silver nanoparticles were directly obtained from the TEM image using the Image J software, a Java program developed by the National Institute of Mental Health, Bethesda, Maryland (MD), USA.

### 4.3. Determination of the Minimum Inhibitory Concentration (MIC) and the Minimum Bactericidal Concentration (MBC)

The MICs and MBCs of the antibiotics (CAZ, IMI, MER, and GENT) and AgNPs were determined by the plate count method measured by serial dilutions as previously described [37]. Briefly, a range of concentrations of AgNPs (4–64 μg/mL) and the antibiotics (0.25–1024 μg/mL) was prepared in a 96-well plate by serial dilution. The solutions were then added to an equal volume of the bacterial suspension (100 μL) in each well of a 96-well plate, with the final cell concentration ranging from 10^6^ to 10^7^ CFU/mL. The plates were incubated at 37 °C for 24 h. Then, 50 μL of each treated solution were collected for a serial tenfold dilution plate count with sterile water, and 10 μL of each dilution were dropped on Mueller Hinton agar in triplicate and incubated overnight at 37 °C to count the bacterial colonies formed. Bacteria with no treatment were used as the control. The MIC was defined as the lowest concentration that inhibits 99% of bacterial growth, and the MBC was defined as the lowest concentration that inhibits 100% of the bacterial growth. The percent inhibition was calculated using the following formula: (1 − (CFU sample/CFU control)) × 100.

### 4.4. Determination of Synergistic Antibacterial Effects

The synergistic antibacterial effects were evaluated using the FIC index and the FBC index, which were obtained by plate counting using the microdilution checkerboard method. Briefly, a range of concentrations of AgNPs (4–64 μg/mL) and the antibiotics (0.5–1024 μg/mL) was prepared by serial dilution, and then 50 µL of each sample of AgNP and antibiotic concentration were transferred to each well of a 96-well plate (total 100 µL of each antibiotic–AgNPs combination). After that, 100 µL of the cell suspension of each bacterial isolate (final cell concentration range of 10^6^–10^7^ cells/mL) were added in each well of the 96-well plate containing the antibiotic–AgNPs mixture. The plates were incubated at 37 °C for 24 h. Due to the inherent absorbance of the silver solution, we needed to determine antimicrobial activity using the plate count method. After 24 h of incubation, 50 μL of each treated condition were collected for a serial 10-fold dilution plate count with sterile water in triplicate for the determination of the MIC and the MBC.

The FIC or FBC index was calculated to evaluate the combined antimicrobial effect of the antibiotics and AgNPs:

$$FICI=\frac{MIC\;of\;drug\;A\;in\;the\;combination}{MIC\;of\;drug\;A\;alone}+\frac{MIC\;of\;drug\;B\;in\;the\;combination}{MIC\;of\;drug\;B\;alone}$$

$$FBCI=\frac{MBC\;of\;drug\;A\;in\;the\;combination}{MBC\;of\;drug\;A\;alone}+\frac{MBC\;of\;drug\;B\;in\;the\;combination}{MBC\;of\;drug\;B\;alone}$$

Synergy, antagonism, and indifference were defined as the FIC or FBC indices ≤ 0.5, >4, and >0.5 and ≤4, respectively [72].

### 4.5. Bacterial Growth Curve of an Antibiotic with AgNPs Combination

Mid-log-phase cultures of *B. pseudomallei* 1026b were prepared in MHB broth with the final cell concentration ranging from 10^6^ to 10^7^ CFU/mL. Then, the bacteria were incubated with CAZ, GENT, the combination of CAZ or GENT with AgNPs at the MIC, MBC, FIC, and FBC in a 96-well plate. The plate was measured at 630 nm every 30 min for 24 h using a microplate reader (UT-2100C, MRC Ltd-laboratory equipment, Holon, Israel). The bacteria without adding agents were used as the positive control.

### 4.6. Killing Kinetic Assay

Kinetic changes in the antimicrobial effect of each combination were examined by the serial dilution method. *B. pseudomallei* 1026b at the inoculum of 10^6^–10^7^ CFU/mL were treated with AgNPs, the antibiotics, and the antibiotic–AgNPs combinations at the final concentration equal to the MIC, MBC, FIC, or FBC. The bacteria were incubated at 37 °C using a shaking incubator at 180 rpm. At 0, 0.5, 1, 3, 6, and 24 h of incubation time, 50 μL of each treated condition were collected for plate counts in triplicate. The bactericidal effect was defined as a ≥3 log_10_ reduction in the CFU/mL compared with the initial inoculum.

### 4.7. Evaluating the Morphological Changes of the Bacterial Cells

The morphological changes of the treated bacterial cells were observed using scanning electron microscopy (SEM). The colonies of *B. pseudomallei* 1026b were grown in MHB for 24 h at 37 °C and then subcultured in 10 mL of the same medium for 3 h to yield a mid-logarithmic growth phase culture. Subsequently, the bacteria were washed three times with deionized water and resuspended in the same solution to the final concentration of 1 × 10^7^ CFU/mL. The cells were treated with AgNPs or the antibiotics alone or the antibiotic–AgNPs combinations at the MIC and FIC for 5 h and at the FBC for 1 h, respectively. All the cells were washed two times with deionized water and then fixed with 2.5% glutaraldehyde for 1 h and dehydrated in a gradient of ethanol (30%, 50%, 70%, and 90%) for 10 min followed by rinsing in 100% ethanol twice. The cells were coated with gold and observed by scanning electron microscopy (LEO 1450VP, Carl Zeiss AG, Oberkochen, Germany) [73].

## 5. Conclusions

Due to the increasing problem of antibiotic resistance, *B. pseudomallei* infections have become harder to treat. To address this problem, we offer alternative ways to potentially combat the bacteria. In this study, we evaluated the synergism of antibiotics with AgNPs against *B. pseudomallei*, which has not been previously reported. *B. pseudomallei* was susceptible to IMI, MER, and AgNPs but was completely resistant to GENT. We found that only *B. pseudomallei* 316c was resistant to CAZ, an antibiotic recommended as the first-line therapy for severe melioidosis. We then combined CAZ, IMI, MER, and GENT with AgNPs and found that the combinations presented synergistic or indifferent effects, but no antagonism was found against all the three isolates of the *B. pseudomallei* tested. Additionally, a combination of an antibiotic with AgNPs can improve kinetic killing of bacteria to shorten the time. In correlation with the results provided in this work, we concluded that certain combinations of antibiotics with AgNPs are able to enhance the antimicrobial effect of antibiotics by reducing the antibiotic dose that is needed for bacterial growth inhibition. AgNPs showed strongly enhanced bactericidal activity and restored bactericidal activity of inactive antibiotics (CAZ and GENT) against *B. pseudomallei*. These findings support the study of antibiotic–AgNPs combinations as an alternative design strategy for new therapeutics to more effectively overcome melioidosis. Clinically, these mixtures of antibiotics with AgNPs could be used through topical routes of administration for skin abrasion exposure to *B. pseudomallei*. Furthermore, given the optimal distribution of AgNPs administered intravenously, preformed mixtures of these antibiotics with AgNPs could also be given through the IV route, as is the standard of care with CAZ for septicemic melioidosis. While further in vivo characterizations of efficacy and toxicity are necessary in future works, these results clearly demonstrate the potential for restoring potency of antibiotics against *B. pseudomallei* through their combination with AgNPs and provide an important tool for overcoming drug resistance as new CAZ-resistant strains continue to rise.

## Figures and Tables

**Table 1:** Minimum inhibitory concentrations (MICs) and minimum bactericidal concentrations (MBCs) of antibiotics and AgNPs against *B. pseudomallei* in three clinical isolates (serial dilution plate count method). The data represent the average of three independent experiments which gave identical values.

Bacteria (Isolates)	MIC (μg/mL)					MBC (μg/mL)				
	CAZ	IMI	MER	GENT	AgNPs	CAZ	IMI	MER	GENT	AgNPs
* 1026b	4	1	2	32	8	64	2	4	512	16
** H777	8	1	2	16	16	32	2	4	256	32
*** 316c	128	0.5	2	64	16	512	1	4	512	32

The MIC value is the lowest concentration that inhibits ≥ 99% of bacterial growth, and the MBC value is the lowest concentration that inhibits 100% of the bacterial growth. CAZ, ceftazidime; IMI, imipenem; MER, meropenem; GENT, gentamicin; AgNPs, silver nanoparticles. * CAZ-nonresistant isolate; ** moderately CAZ-resistant isolate; *** highly CAZ-resistant isolate.

**Table 2:** Minimum inhibitory concentrations (MICs; μg/mL) of the antibiotics alone or in combination with AgNPs. Fold change in the MIC of the antibiotics in combination or alone.

Antibiotics	*B. pseudomallei* Isolates	MIC (µg/mL)		Decrease in the Concentration (-Fold)	FICI	Type of Interaction
		Alone	Combination			
Ceftazidime	1026b	4	1 (2)	4	0.5	S
	H777	8	2 (2)	4	0.375	S
	316c	128	16 (4)	8	0.375	S
Imipenem	1026b	1	0.5 (2)	2	0.75	I
	H777	1	0.25 (4)	4	0.5	S
	316c	0.5	0.25 (4)	2	0.75	I
Meropenem	1026b	2	0.5 (2)	4	0.5	S
	H777	2	0.5 (2)	4	0.375	S
	316c	2	0.5 (2)	4	0.375	S
Gentamicin	1026b	32	2 (2)	16	0.312	S
	H777	16	4 (4)	4	0.5	S
	316c	64	16 (2)	4	0.375	S

Synergy (S) was defined as an FIC index ≤ 0.5; antagonism (A) was defined as an FIC index > 4; and indifference (I) was defined as an FIC index > 0.5 and ≤4. The FIC index is the fractional inhibitory concentration; (2) and (4) represent the concentrations of AgNPs in combination with antibiotics of 2 μg/mL and 4 μg/mL, respectively. The data represent three independent experiments that all gave identical values, and no standard deviation was observed.

**Table 3:** Minimum bactericidal concentrations (MBCs; μg/mL) of the antibiotics alone or in combination with AgNPs. Fold change in the MBC of the antibiotics in combination or alone.

Antibiotic	*B. pseudomallei* Isolates	MBC (µg/mL)		Decrease in the Concentration (-Fold)	FBCI	Type of Interaction
		Alone	Combination			
Ceftazidime	1026b	64	4 (4)	16	0.313	S
	H777	32	8 (4)	4	0.375	S
	316c	512	16 (8)	32	0.281	S
Imipenem	1026b	2	0.5 (4)	4	0.5	S
	H777	2	0.5 (8)	4	0.5	S
	316c	1	0.25 (4)	2	0.625	I
Meropenem	1026b	4	1 (2)	4	0.375	S
	H777	4	1 (4)	4	0.375	S
	316c	4	1 (4)	4	0.375	S
Gentamicin	1026b	512	1 (4)	512	0.252	S
	H777	256	4 (8)	64	0.265	S
	316c	512	16 (8)	32	0.289	S

Synergy (S) was defined as an FBC index ≤ 0.5; antagonism (A) was defined as an FBC index > 4; and indifference (I) was defined as an FBC index > 0.5 and ≤4. The FBC index is the fractional bactericidal concentration; (2), (4), and (8) represent the concentrations of AgNPs in combination with antibiotics of 2 μg/mL, 4 μg/mL, and 8 μg/mL, respectively. The data represent three independent experiments that all gave identical values, and no standard deviation was observed.

## Data Availability

The data presented in this study are available upon request from the corresponding author.
